# The cerebral mechanism of acupuncture for chronic insomnia with gastrointestinal disorder: protocol for a randomized controlled trial

**DOI:** 10.1186/s13063-021-05332-3

**Published:** 2021-06-07

**Authors:** Wei Peng, Xiaojuan Hong, Yaru Huangfu, Zhao Sun, Wei Shen, Fen Feng, Liang Gong, Zhifu Shen, Baojun Guo, Leixiao Zhang, Yanan Wang, Ying Zhao, Tianmin Zhu, Youping Hu, Siyi Yu

**Affiliations:** 1 Acupuncture & Tuina College, Chengdu University of Traditional Chinese Medicine, Chengdu, 610075 Sichuan China; 2 Hainan Medical University, Haikou, 571199 Hainan China; 3 Affiliated Hospital of Chengdu University of Traditional Chinese Medicine, Chengdu, 610072 Sichuan China; 4 Chengdu Second People’s Hospital, Chengdu, 610017 Sichuan China; 5 North Sichuan Medical College, Nanchong, 637100 Sichuan China; 6 Henan Provincial People’s Hospital, Zhengzhou, 450003 Henan China; 7 Rehabilitation and Health Preservation College, Chengdu University of Traditional Chinese Medicine, Chengdu, 610075 Sichuan China

**Keywords:** Chronic insomnia disorder, Gastrointestinal disorder, Multimodal magnetic resonance imaging, Acupuncture

## Abstract

**Background:**

Many patients with chronic insomnia disorder (CID) have gastrointestinal (GI) symptoms. First-line insomnia medications do not treat GI problems. Acupuncture has a comprehensive regulative action on both CID and GI disorder and is receiving increasing attention. Recent studies indicate that both CID and GI diseases may cause abnormal brain activity. However, the neurological mechanism underlying the effect of acupuncture on such diseases is still unclear. The aim of this study is to explore the pathological mechanisms of CID with GI discomfort, as well as the main response characteristics of acupuncture treatment from multiple perspectives using multimodal magnetic resonance imaging (MRI).

**Methods:**

A total of 60 participants with CID and GI disorders will be randomly divided into two groups (real acupuncture group and sham acupuncture group; ratio of 1:1). Patients will receive 20 sessions (five sessions per week) of real acupuncture treatment or sham acupuncture treatment. The primary outcome is the aggregate score on the Pittsburgh Sleep Quality Index. Secondary outcomes are scores on the Gastrointestinal Symptom Rating Scale, Self-Rating Anxiety Scale, and Self-Rating Depression Scale. Multimodal MRI scans and clinical assessments will be performed both at baseline and post-treatment. Another 30 age-, sex-, and education-matched healthy subjects will be recruited as controls and will receive MRI scans and clinical evaluations.

**Discussion:**

This study aims to provide scientific evidence for the mechanism of acupuncture in treating CID with GI disorder using multimodal MRI imaging data on brain structure, function, and metabolism.

**Trial registration:**

Chinese Clinical Trial Registry, ChiCTR1800017092 (URL: http://www.chictr.org.cn/showproj.aspx?proj=27173). Registered on July 11, 2018.

**Supplementary Information:**

The online version contains supplementary material available at 10.1186/s13063-021-05332-3.

## Background

Chronic insomnia disorder (CID) is defined as difficulty initiating or maintaining sleep that is associated with daytime consequences and occurs at least three times per week for more than 3 months; CID has a prevalence rate of 5% to 10% [1]. Typical nighttime symptoms include difficulty falling asleep, difficulty maintaining sleep, and waking early [2, 3]. Additional symptoms include fatigue, memory decline, cognitive deficits, and mood disorder [4, 5]. Recent studies have identified a correlation between sleep disturbances and gastrointestinal (GI) diseases [6]. According to a previous survey [2], about 27.5%–33.4% of insomnia patients have concomitant GI symptoms, such as dyspepsia, diarrhea, and constipation. Interestingly, patients with multiple GI symptoms are more likely to have sleep problems [7–9]. It has recently been suggested that GI function plays a vital role in sleep [10]. These findings provide new insights into the pathological features and treatment of CID associated with GI.

Pharmacotherapy is the primary recommended treatment for CID, according to the American Academy of Sleep Medicine clinical practice guideline [1]. Although there is evidence for the short-term efficacy of insomnia medications [11, 12], long-term use is associated with the risk of addiction and adverse drug reactions, particularly GI side effects [13]. Therefore, treatments with fewer side effects are needed for this population. Acupuncture is a possible alternative therapy. Multiple studies have shown that acupuncture effectively improves sleep quality [14, 15]. Other studies have found that acupuncture can improve inflammation in gastrointestinal diseases and promote the recovery of gastrointestinal function [16–18]. Moreover, acupuncture is more effective than drug therapy in treating insomnia with GI disorder [19, 20]. However, the mechanism underlying the effect of acupuncture on CID with GI symptoms remains to be elucidated.

In recent years, magnetic resonance imaging (MRI) techniques, such as functional magnetic resonance imaging (fMRI), structural magnetic resonance imaging (sMRI), diffusion tensor imaging (DTI), and magnetic resonance spectroscopy (MRS), have been widely used to study the pathophysiological mechanisms of insomnia. Using fMRI, we previously confirmed that the core pathological features of patients with CID are evident in the structural and functional plasticity of the brain [21–24]. DTI technique is currently the only non-invasive method to effectively observe and track white matter fiber tracts. One study found that compared with healthy people, the fractional anisotropy (FA) value of the anterior internal capsule in insomniacs decreased [25]. Another study also found that the diffusion properties of the right arcuate fasciculus and the superior longitudinal fasciculus, such as the values of FA, mean diffusivity (MD), radial diffusivity (RD), and axial diffusivity (AD), were changed in insomnia patients [26]. By analyzing the diffusion properties of white matter, we can infer the influence mechanism of acupuncture on the white matter of CID patients with GI disorder. MRS is the only non-invasive method that can quantify biochemical substances in the brain [27], and MRS studies have identified abnormal brain metabolic activity in insomnia patients [28, 29]. Such studies have helped to clarify the pathology and treatment mechanisms of CID with GI disorder.

There is growing evidence from MRI studies that acupuncture can restore abnormal brain structure, connections, and metabolism in pathological states [30–32]. Both insomnia [33] and GI disease [34] studies have shown that acupuncture can repair abnormal connections in the brain. Previous studies on the mechanism of acupuncture in treating CID have shown that acupuncture affects the activation of brain regions through tactile somatosensory specific stimuli [35, 36]. Acupuncture can regulate the activity of specific brain regions related to sleep experience, such as the prefrontal lobe, temporal lobe, parietal lobule, anterior cingulate, supramarginal gyrus, and precuneus [36–38]. In addition, acupuncture can also regulate cerebral blood flow and neurotransmitter levels such as 5-hydroxytryptamine (5-HT), gamma aminobutyric acid/glutamate (GABA/Glu) in the brain of patients with insomnia [39, 40]. At present, neuroimaging technology has become the mainstream means to study the mechanism of acupuncture treatment of CID. However, no studies have focused on the mechanism underlying the effect of acupuncture on CID with GI disorder. Therefore, a multimodal MRI approach combining fMRI, sMRI, DTI, and MRS is needed to explore the main mechanisms by which acupuncture affects CID with GI disorders.

For these reasons, we designed this multimodal neuroimaging trial. The study aims are as follows: (1) to confirm the efficacy of acupuncture for patients with insomnia and GI discomfort by comparing it with sham acupuncture; (2) to explore the central response mechanism underlying the effect of acupuncture on CID with GI disorders, focusing on cerebral structure, function, and metabolism; (3) to explore a possible correlation between brain activity changes and symptom improvement caused by different acupuncture modes.

## Methods/design

The protocol was developed in accordance with Standard Protocol Items for Clinical Trials with Traditional Chinese Medicine 2018 [41] and Standards for Reporting Interventions in Clinical Trials of Acupuncture [42].

### Study design

**Fig. 1:**
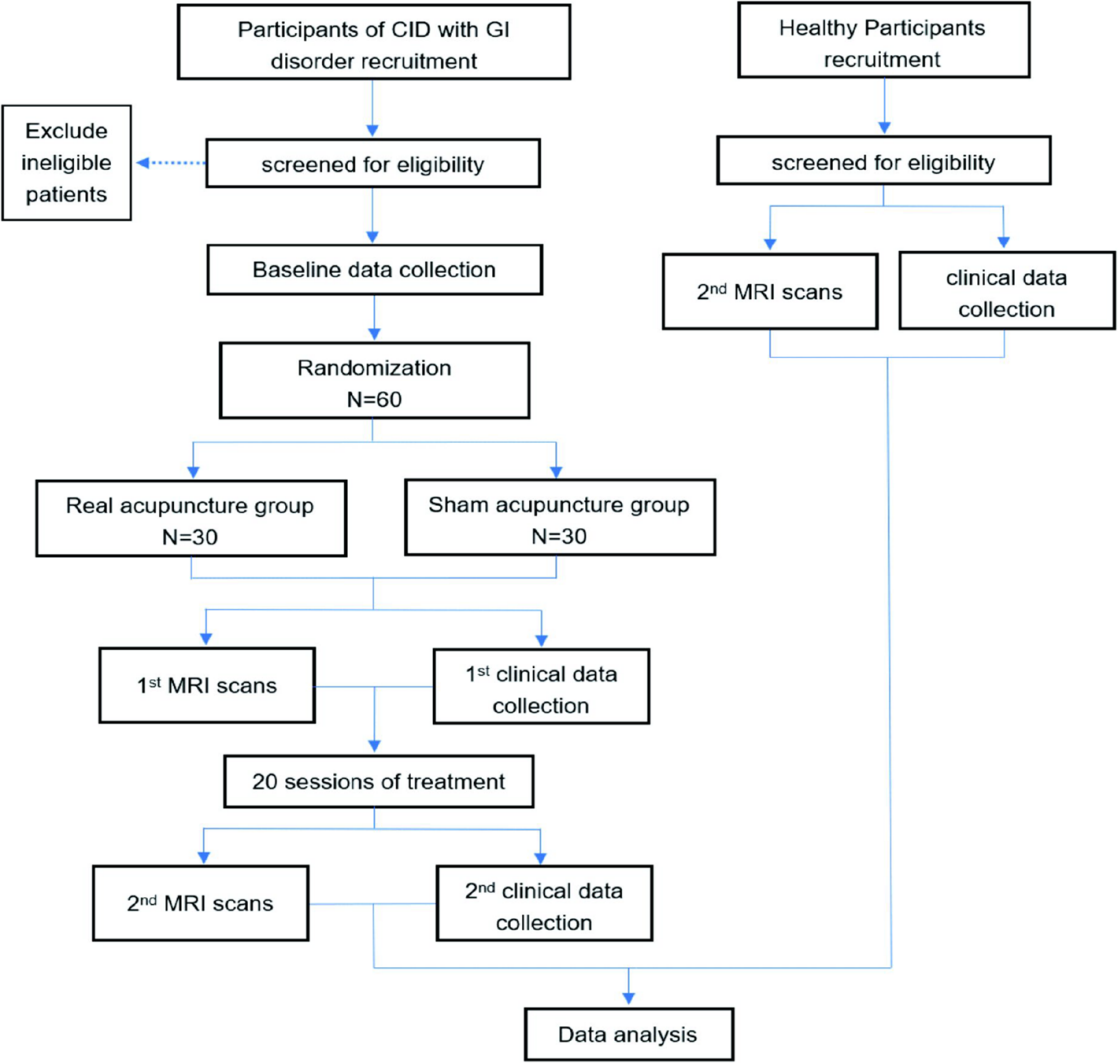
Flowchart of the trial. CID, chronic insomnia disorder; GI, gastrointestinal; MRI, magnetic resonance imaging

**Fig. 2:**
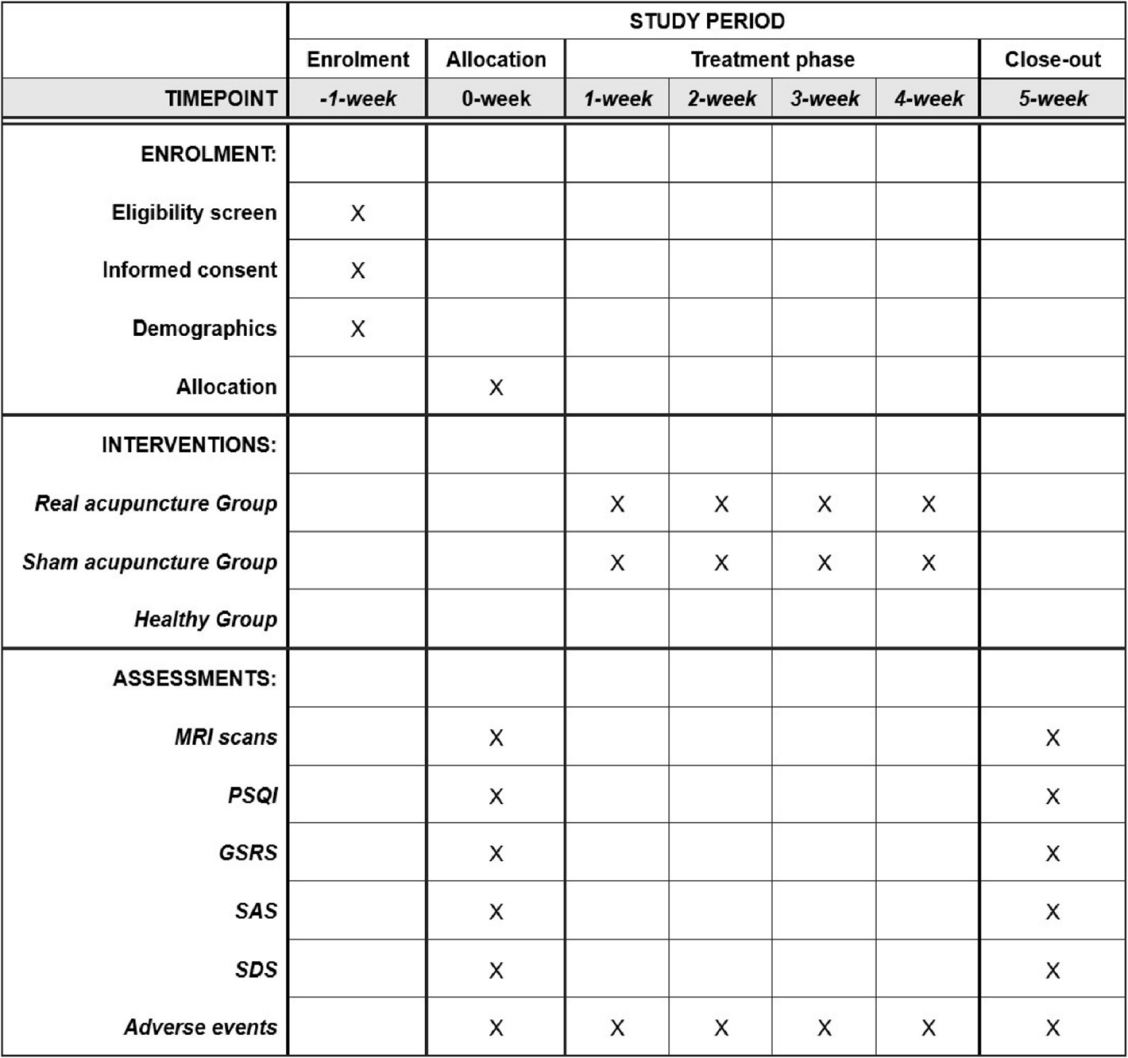
Time schedule of enrolment, interventions and assessments. Eligible patients will be screened according to inclusion and exclusion criteria. These patients will then sign an informed consent form and undergo a medical examination. Eligible insomniacs will be randomly assigned to either the true or sham acupuncture groups and complete clinical and MRI assessments before treatment. After a 4-week treatment period, these patients will undergo clinical and MRI assessments again. In addition, adverse events will be recorded in the CRFs at any time during treatment. MRI, magnetic resonance imaging; PSQI, Pittsburgh sleep quality index; GSRS, gastrointestinal symptom rating scale; SAS, self-rating anxiety scale; SDS, self-rating depression scale

This randomized, single-blind, sham-controlled neuroimaging study will be conducted at the affiliated hospital of Chengdu University of Traditional Chinese Medicine (CDUTCM), China. A total of 60 CID patients with GI disorder will be recruited using advertisements on posters and hospital networks. All patients meeting the inclusion criteria will randomly receive either 20 sessions of real acupuncture treatment or 20 sessions of sham acupuncture treatment. A multimodal MRI examination and evaluation will be performed at both baseline and the end of treatment. Additionally, 30 healthy controls (HCs) matched for age, sex, and education level will receive one multimodal MRI scan and a clinical assessment. A flowchart of the trial design is shown in Fig. 1, and the participant timeline is shown in Fig. 2.

#### Recruitment

Study participants will be recruited mainly from outpatient clinics in major hospitals in Chengdu. We will post the recruitment advertisements approved by the ETHICS committee on the poster boards of each hospital and promote them through WeChat software (Tencent Inc., Shenzhen, China). The entire recruitment process will be conducted by researchers (YN Wang and Y Zhao) who are not involved in the treatment process.

#### Inclusion criteria

Patients diagnosed with CID are eligible to participate in the study. Inclusion criteria are (1) aged between 18 and 65 years, right-handed, any gender; (2) meets the CID diagnostic criteria in the International Classification of Sleep Disorders-Third Edition (ICSD-3) [43], and meets the criteria for spleen and stomach disharmony syndrome in the Clinical Terminology of Traditional Chinese Medical Diagnosis and Treatment—Syndromes (GB/T 16751.2-1997) [44]; (3) Pittsburgh Sleep Quality Index score above 7 (PSQI score > 7); (4) not taking any medication or health care products to improve sleep quality and experienced GI diseases at least 2 weeks before study enrollment; (5) has not participated in other clinical trials within the last month; and (6) agrees to voluntarily participate in the study and signs an informed consent form.

The HCs must meet the following inclusion criteria: (1) passes the neuropsychological tests and reports good sleep quality; (2) no GI symptoms; (3) all physiological indexes within the normal range following a physical examination, and no previous functional or organic disease or head injury; and (4) agrees to voluntarily participate in the study and signs an informed consent form.

#### Exclusion criteria

Patients with any one of the following criteria will be excluded: (1) any severe conditions of the cardiovascular, cerebrovascular, liver, kidney, and hematopoietic systems; (2) secondary insomnia caused by drugs, cervical spondylosis, or other diseases; (3) history of psychiatric and neurological disorders or head trauma with loss of consciousness; (4) abuse of psychotropic drugs for anxiety and depression or sedative and hypnotic drugs; (5) pregnant, preparing for pregnancy, or lactating; and (6) any MRI contraindications such as claustrophobia, cardiac pacemaker, defibrillator, heart stenting, metal dentures, or intrauterine device.

The exclusion criteria for the HC are the same as for the patients.

#### Withdrawal criteria

Participants with any of the following conditions will be withdrawn: (1) complications that affect safety, (2) serious adverse events, and (3) unwilling to follow the study protocol for examination and treatment.

### Sample size

According to the previous studies [45, 46], the mean difference of PSQI scores between acupuncture and sham acupuncture was at least 2.7. We expect the mean PSQI score to decrease by 4.5 points in the acupuncture group and 1.8 points in the control group after treatment. When α was 0.05, 1-β was 0.9, and standard deviation was 3, the calculated sample size was 27. Given that this is a neuroimaging study, sample size requirements in the neuroimaging field should also be taken into account. At present, there is no accepted standard for the sample size calculation of imaging studies. The requirements of imaging studies [47, 48] and previous similar studies [49, 50] suggest that 12–26 individuals in each group is reasonable sample size for data analysis. To allow for potential problems such as subject dropout and invalid imaging data owing to head movements, a sample of 30 patients in each group is considered reasonable.

### Randomization and allocation concealment

A researcher (ZF Shen), who will not be involved in the process of patient recruitment and treatment, will use SAS 9.2 software (SAS Institute Inc., Cary, NC, USA) to generate 60 random numbers and 60 serial numbers. Then, he will divide them into two groups based on random numbers and get the group numbers. These random numbers, serial numbers, and group numbers will be placed in opaque, sealed envelopes. Each sealed envelope will be numbered in sequence and kept in a safe place until the study is complete. If participants met the inclusion and exclusion criteria, envelopes with the same serial number will be opened in the order of enrollment by the same researcher (ZF Shen) and the treatment allocation results will be forwarded to the acupuncturists. Except for the acupuncturists and the researchers who opened the envelopes, neither the participants nor the other researchers would know the participants' groups.

### Blinding

The blinding of acupuncturists is quite difficult to achieve [51]. Patients will be told that they would be randomly assigned to one of two effective interventions after enrollment. During the treatment, patients will receive treatment in acupuncture treatment rooms with compartments to avoid relevant communication between patients. Acupuncturists will also be required to keep treatment plans secret. The names of the treatment and control groups will be replaced with meaningless letter combinations to prevent outcome assessors and statisticians from knowing how patients are grouped. Therefore, only patients, outcome assessors, and statisticians will be fully blind in this study.

### Interventions

Treatments will be performed by certified acupuncturists with at least 3 years of acupuncture experience. Disposable sterile filiform needles (XingLin acupuncture needle, Φ0.25 × 25 mm/Φ0.25 × 40 mm, Tianjin Yi Peng Medical Instrument Co., Ltd., China) will be used. Both the acupuncture and sham acupuncture treatments will comprise 20 sessions over 4 weeks (five sessions per week).

#### Real acupuncture group

**Fig. 3:**
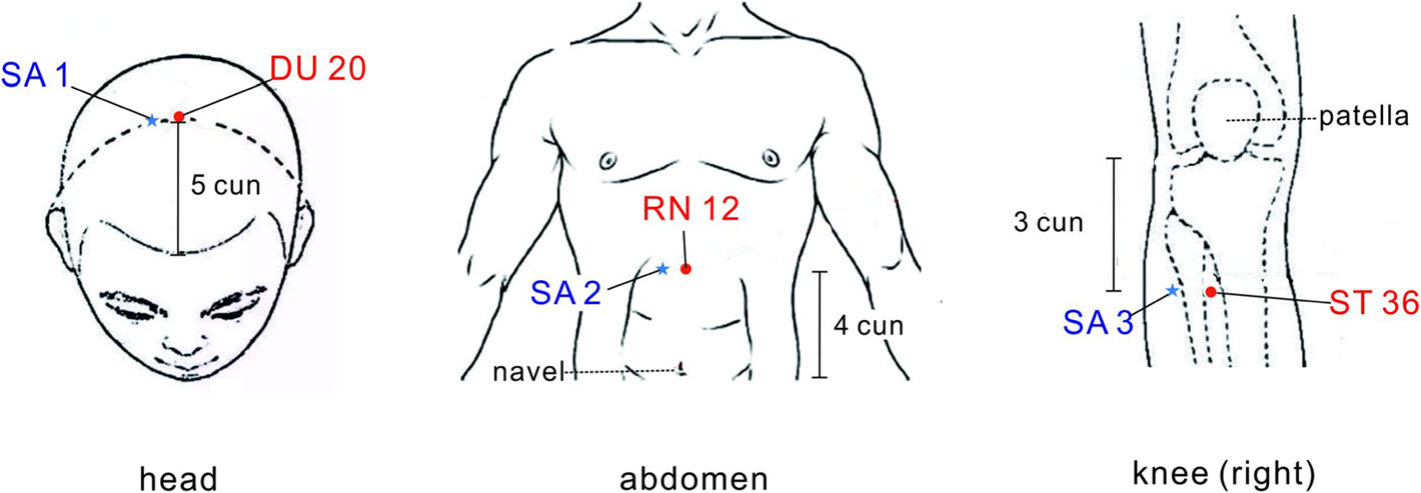
Location of acupoints and sham acupoints. SA, sham acupoint

Patients in the real acupuncture group will receive acupuncture treatment on the acupoints *Baihui* (DU20), *Zhongwan* (RN12), and *Zusanli* (ST36) with disposable sterile filiform needles. All acupoints will be located according to the World Health Organization Standard Acupuncture Locations and are shown in Table 1 and Fig. 3. Needles in DU20 will be inserted 0.5–1 cun (a unit of measurement in acupuncture, 1 cun = 25 mm) using horizontal needling. Needles in RN12 and ST36 will be inserted 0.5–1.5 cun using perpendicular needling after skin disinfection. After the needle enters into the skin, needles will be manipulated by twirling, lifting, and thrusting to generate the sensation of de qi in the local tissue. The experience of de qi is characterized by sensations of numbness, heaviness, or distension felt by the patient. All needles will be retained for 30 min.

**Table 1:** Acupoint locations for the real acupuncture group

Acupoints	Locations
*Baihui* (DU20)	The intersection of the crown midline and the apex of the ears
*Zhongwan* (RN12)	On the upper abdomen, anterior median line, four cun^a^ above the navel
*Zusanli* (ST36)	On the outside of the calf, three cun below *Dubi* (ST35)^b^

^a^According to the theory of traditional Chinese medicine, the navel is 8 cun below the xiphoid process

^b^ST35 is located on the lateral depression of the patellar ligament

#### Sham acupuncture group

The sham acupuncture group will receive a superficial skin penetration treatment at sham acupoints (SA). These SA are located near real acupoints (2 cm lateral from DU20, RN12, and ST36) but do not belong to any known meridian and are not conventional acupoints. The SA locations are shown in Table 2 and Fig. 3.

**Table 2:** Sham acupoints locations for the sham acupuncture group

Sham acupoints (SA)	Locations
`SA 1	2 cm to the right of DU20
SA 2	2 cm to the right of RN12
SA 3	2 cm outside of ST36

cm, centimeter

Superficial skin penetration at SA, without needle manipulation for de qi, is a common sham acupuncture method used in many acupuncture randomized controlled trials [52]. Patients in this group will undergo an acupuncture procedure similar to the one received by patients in the real acupuncture group.

### Concomitant medication

Patients will be advised to avoid medication as much as possible during the study. Following medical ethical principles, if patients’ insomnia symptoms do not significantly improve and they cannot tolerate their insomnia, they may temporarily take sleeping drugs. Except for sleeping drugs, other drugs will be not allowed to be used in the trial. Researchers will record the name, dosage, and time of the drugs taken in detail in the Case Report Form (CRF).

### Multimodal MRI data acquisition

MRI examinations will be performed at the MRI Center at the University of Electronic Science and Technology of China using a 3.0 T MRI scanner (GE Discovery MR750, USA) equipped with a standard 8-channel head coil. MRI scans will be assessed at baseline and after 4 weeks. Subjects will be told to eat light food; avoid drinking strong tea, coffee, and wine; and avoid strenuous exercise the day before the scan. To prevent head movements affecting the image, foam pads and towels will be used to hold the head. During the MRI examination, all subjects will wear earplugs and be instructed to relax with their eyes closed and keep their heads clear. The scanning procedure produces a three-dimensional T1 image (3D-T1), a blood oxygenation level-dependent fMRI (BOLD-fMRI), a DTI sequence, and a proton ^1^H-MRS. The 3D-T1 scanning parameters will be as follows: repetition time (TR)/echo time (TE) = 5.988/1.972 ms, slice thickness = 1 mm, slice number = 154, field of view (FOV) = 256 × 256 mm. The BOLD-fMRI scanning parameters will be as follows: TR/TE = 2000/30 ms, flip angle = 90°, slice number = 35, matrix size = 3.75 × 3.75, FOV = 64 × 64 mm, slice thickness = 4 mm. The DTI data will be obtained using the following parameters: FOV = 240 × 240 mm, TR/TE = 6800/93 ms, matrix size = 128 × 128, and slice thickness = 3 mm with no gap. The proton ^1^H-MRS asymmetric PRESS sequence includes TE1 = 25 ms, TE2 = 85 ms, TR = 2 s, and a 2 × 2 × 2 cm area of interest on the anterior cingulate gyrus will be selected [29].

### Outcome measures

The outcome assessments will be performed at baseline and after 20 treatment sessions. All outcome assessors will be trained in conducting interviews and performing measurements before the study begins and will follow a standard protocol. An overview of the outcome measurement at different time points is shown in Fig. 2.

#### Primary outcomes

The primary outcomes include MRI indicators and PSQI scale score. This study will focus on the following MRI indicators: the amplitude of low-frequency fluctuation (ALFF), seed-based functional connectivity (FC), cortical thickness, AD, FA, MD, RD, and Glu. ALFF and FC are the most common analysis indicators in fMRI, and their changes can reflect the functional activities of the brain [53]. Cortical thickness is a quantitative index of cortical morphology, which can directly reflect the changes of brain structure [54]. AD, FA, MD, and RD are all important indexes in DTI technology, which can be used to observe the integrity and connectivity of organizational structure [55]. Glu is an excitatory neurotransmitter involved in the occurrence and maintenance of sleep and wakefulness [56].

The PSQI comprises 19 self-evaluation items, among which 18 items generate 7 components. Each component is graded from 0 to 3, and the cumulative score of each component is the total score of PSQI, which ranges from 0 to 21. The PSQI is the most widely used sleep quality rating scale [57].

#### Secondary outcomes

The Gastrointestinal Symptom Rating Scale (GSRS) [58] will be used to measure GI symptoms.

The Self-Rating Anxiety Scale (SAS) and the Self-Rating Depression Scale (SDS) [59] will be used to assess subjects’ emotional state.

### Data management

Clinical data will be managed using printed and electronic CRF accessible only by the research team. The CRF is the original record and cannot be changed at all. It will be kept by a researcher of the research team. At the end of the trial, two researchers will enter data from the CRF into a computer. The Evidence-based Medicine Center of the CDUTCM will regularly monitor the trial data.

### Data analysis

#### Clinical data analysis

Statistical analyses of the clinical data will be performed using SPSS 20.0 statistical software (IBM Corporation, Armonk, NY, USA) and supervised by a skilled statistician blinded to group allocation. The Kolmogorov–Smirnov test will be used to test the normal distribution of continuous variables. Continuous normally distributed data will be reported as means and standard deviations; continuous non-normally distributed data will be expressed as medians with interquartile ranges. Categorical data will be presented as frequencies or percentages.

For the demographic and clinical information at baseline, the chi-squared test or Fisher’s exact test will be used for comparison of dichotomous data. The two-sample t test will be used for normally distributed continuous data or the Mann-Whitney U test for non-normally distributed data. To compare baseline and post-treatment changes in the same group, we will use the paired t test. Covariance analysis using baseline data as covariates will be used to compare the effects of different interventions on continuous data. In addition, gender differences and age differences may influence the data results. Therefore, in order to avoid the influence of gender, age and other factors on the data, we will also adopt covariance analysis, taking gender, age, and other factors as covariates respectively, and conduct covariance analysis with continuous data. A p value < 0.05 will be considered statistically significant.

#### Imaging data analysis

The fMRI data will be preprocessed and analyzed using SPM12 (http://www.fil.ion.ucl.ac.uk/spm/) and CONN toolbox 18b (https://web.conn-toolbox.org/) with MATLAB 2014b (MathWorks, Inc., Natick, MA, USA). The sMRI data will be analyzed using the VBM toolbox within SPM12. DTI data will be processed using FSL Software on Linux. MRS data will be preprocessed using the commercially available software LCModel spectral-fitting package (version 6.3-1 N; Stephen Provencher, Inc., Oakville, ON, Canada). After standard preprocessing of each imaging modality, the following brain activity information will be calculated to examine the neural response to different treatments: the amplitude of low-frequency fluctuation, group independent component analyses, seed-based functional connectivity, cortical thickness, tract-based spatial statistics, and Glu. Finally, Pearson correlation analysis will be used to assess the association between changes in multimodal neuroimaging features and improvements in clinical outcomes in each group. MRI data from patients who received the drugs will be excluded because of the effects of the combination on brain function during the trial.

### Patient safety

During treatment, any adverse events (AEs) will be monitored and recorded in the CRFs throughout the trial. Important AEs in this study would be needle-related AEs, such as bleeding, hematoma, dizziness, infection, neurological symptoms, and fainting. Minor AEs will be treated immediately by the attending acupuncturist. For serious AEs, such as the patient experienced syncope during treatment, the researcher should immediately send the patient to the hospital for emergency treatment, and the research team will also pay appropriate financial compensation to the patient. Any unexpected symptoms during treatment must be recorded in the CRF, regardless of their relationship to the study intervention. For all AEs, the researcher should record them in the CRF and report them to the ethics committee. Severe AEs will be reported to the research ethics committee within 48 h

### Patients and public involvement

Patients and the public were not involved in the design and implementation of the study. They were also not asked to advise on the reporting and dissemination of the results.

### Quality control

To ensure the quality of the clinical trial research project and control the risk, CDUTCM will arrange quality supervisors to supervise the intervention process, informed consent forms, case reports, and data records of the study every 3 months. At the same time, an independent quality management team will be set up within the research team to supervise all aspects of the implementation of this project and regularly write monitoring reports.

## Discussion

Acupuncture is a potential therapy for CID. In recent years, some researchers have used MRI to study the neural mechanisms by which acupuncture treats certain types of insomnia, such as perimenopausal insomnia [60] and shift work sleep disorder [61]. However, there have been few reports on the mechanism of acupuncture in treating insomnia and GI comorbidities. Previous studies have found that acupuncture has a good effect on inflammation of GI diseases [16–18], which also provides strong support for the treatment of CID patients with GI diseases with acupuncture. In our protocol, multimodal MRI will be used to explore the pathological mechanism of CID with GI disorder and the main response characteristics of acupuncture therapy for this type of insomnia. There will be several study strengths and limitations.

In the theoretical system of traditional Chinese medicine, insomnia accompanied by GI disorder is a common syndrome. According to the Yellow Emperor’s Canon of Internal Medicine, a famous Chinese medical text, Chinese doctors discovered a link between GI illness and sleep more than 2,000 years ago, and developed the principle of “harmonizing the stomach and mind” [62]. Our acupoint selection protocol is based on this treatment principle, and the selected points DU20, RN12, and ST36 are commonly used acupoints for insomnia associated with GI disorder in acupuncture clinical trials [63]. DU20 improves sleep, and RN12 and ST36 harmonize GI functions. Evidence from clinical trials using the “harmonizing the stomach and mind” principle has confirmed the effectiveness and safety of acupuncture for CID with GI disorder [19, 64, 65]. These acupoints were also presented in a study protocol on the intestinal flora of acupuncture for insomnia and gastrointestinal diseases published by our team [66].

Multimodal MRI will provide technological data to examine the neuropathological mechanism underlying the effect of acupuncture on CID with GI disorder. We first want to explore neuropathological changes in patients with CID and GI disorder from the perspectives of brain structure, function, and metabolism. Our previous study was a preliminary exploration of the pathogenesis of insomnia (rather than CID with GI disorder) in terms of brain function and structure [23, 24, 67]. Other research teams have confirmed that insomnia patients have abnormal brain metabolism using MRS technology [27–29]. At present, there are no brain imaging studies on patients with CID and GI disorders. Most studies on insomnia have identified the abnormal brain regions in CID patients as the anterior cingulate gyrus, hippocampus, thalamus, and other regions [68, 69]; these brain regions also feature in GI imaging studies [70]. It remains to be determined whether the structure, function, and metabolism of these brain regions are also changed in CID with GI disorder. Second, we wish to explore the central response mechanism of acupuncture. There is evidence that acupuncture can regulate the neuroplasticity and functional connectivity of abnormal brain regions in other diseases [31, 71], but most studies have used only a single neuroimaging pattern and cannot fully explain the mechanism of acupuncture. Multimodal MRI provides a new way of exploring the mechanism underlying acupuncture. It can examine the mechanism of action in detail from multiple perspectives, which is a more comprehensive approach than the use of single-mode magnetic resonance technology. At present, multimodal MRI has been used to investigate the effect of acupuncture on depression, chronic fatigue syndrome, and other diseases [72–74]. In this study, we will use multimodal MRI combined with sMRI, fMRI, DTI, and MRS. Of these techniques, sMRI and DTI can detect structural changes in different brain regions, fMRI can explore changes in functional connections between different brain regions, and MRS can detect changes in different brain metabolites.

To improve the reliability of results and reduce bias, we will incorporate the following quality control measures. First, the inclusion and exclusion criteria will be strictly applied and the subject groups will be matched on age, gender, height, weight, and other variables. Second, previous studies have found that left-handers and right-handers show different signals in the left and right hemispheres of the brain when performing certain tasks, with more activation in the left hemisphere of the brain in right-handed people [75, 76]. In China, there are more right-handed people than left-handed people. Considering the differences in functional signals between the left and right sides of the brain caused by handedness, this study will only include right-handed people. Third, a project workshop manual will be developed. All the members of our team will be trained to use related diagnostic standards, methods, and evaluation forms. Acupuncture will be performed by two equally qualified acupuncture physicians who will only perform acupuncture after passing a training trial agreed by the research group. Fourth, during the MRI scanning process, the participants’ physiological and mental state may affect the accuracy of the MRI data results [77]. Therefore, we will use SAS and SDS scales to assess the participants' mental state before MRI scanning. In addition, participants will also be told to stop drinking stimulating foods such as alcohol and coffee the day before the MRI scan. Finally, all the imaging will be performed by the same experienced imaging professional.

Some study limitations must be noted. First, during the study, subjects will be allowed to take sleeping medications if they cannot tolerate their insomnia. We cannot ethically limit patients’ choices, and therefore can only ask patients to follow the study treatment regimen as closely as they can, and to record the name and dose of any medication they take on the CRF. Therefore, medication use may be a confounding factor that affects the results. However, as this is a randomized controlled trial, we believe that any noise caused by other treatments will mostly be controlled. Second, the study will be performed in only one center. The bias inherent in the collection and analysis of magnetic resonance data may be increased if the study were to be conducted in multiple locations. Therefore, given the existing technical conditions, we can only conduct a single-center study.

In summary, this study will investigate the effectiveness of acupuncture therapy for CID with GI disorder. We seek to provide scientific evidence for the mechanism of acupuncture in treating CID with GI disorder using multimodal MRI imaging data on brain structure, function, and metabolism.

### Trial status

Study approval was obtained from the Sichuan Traditional Chinese Medicine Regional Ethics Review Committee, China on May 24, 2018 (protocol version number: 20180425). Clinical registration was completed on July 11, 2018 (Chinese Clinical Trial Registry, ChiCTR1800017092). The trial is still ongoing at the time of manuscript submission. The scheduled start of recruitment was 1 August 2018. The anticipated end of recruitment is August 1, 2020.

## Supplementary Information

**Additional file 1.** Standard Protocol Items: Recommendations for Interventional Trials (SPIRIT) 2013 Checklist: Recommended items to address in a clinical trial protocol and related documents.

## Data Availability

Not applicable; no data have yet been generated.

## Abbreviations

AD: Axial diffusivity
AEs: Adverse events
BOLD-fMRI: Blood oxygenation level-dependent functional magnetic resonance imaging
CID: Chronic insomnia disorder
CRF: Case report form
DTI: Diffusion tensor imaging
FA: Fractional anisotropy
FC: Functional connectivity
FOV: Field of view
GABA: Gamma aminobutyric acid
Glu: Glutamate
GI: Gastrointestinal
GSRS: Gastrointestinal symptom rating scale
HCs: Healthy controls
ICSD-3: International classification of sleep disorders-third edition
MD: Mean diffusivity
MRI: Magnetic resonance imaging
MRS: Magnetic resonance spectroscopy
PSQI: Pittsburgh sleep quality index
RD: Radial diffusivity
SA: Sham acupoints
SAS: Self-rating anxiety scale
SDS: Self-rating depression scale
TR: Repetition time
TE: Echo time
5-HT: 5-Hydroxytryptamine
